# Supplementary material for: Epithelial response to IFN‐γ promotes SARS‐CoV‐2 infection
Source: EMBO Mol Med. 2021 Mar 3;13(4):e13191. doi: 10.15252/emmm.202013191 (PMC7995094; doi:10.15252/emmm.202013191)
Supplement: Supplementary file 1 — Appendix [file EMMM-13-e13191-s002.pdf]

Appendix

Table of content:

1) Appendix Table S1: List of genes with the highest fold-change increase upon infection with SARS-CoV-2 that are also induced by IFN-γ treatment
2) Appendix Table S2: overview of exact p-values and statistical tests

1) Appendix Table S1

GeneSymbol	GeneName	Uninfected vs. Infected Fold Change	Uninfected vs. Infected adj.Pval	Untreated vs. IFN-y treated	Untreated vs. IFN-y treated.adj.Pval
IFI44L	interferon induced protein 44 like	53.09	5.30E-12	41.98	4.90E-12
HRASLS2	HRAS like suppressor 2	42.27	1.72E-12	4.14	4.05E-09
FOLR2	folate receptor beta	42.11	1.93E-08	1.64	0.167484053
RSAD2	radical S-adenosyl methionine domain containing 2	39.38	4.04E-09	13.07	1.27E-07
CXCL10	C-X-C motif chemokine ligand 10	37.01	1.72E-12	63.47	1.28E-13
HERC5	HECT and RLD domain containing E3 ubiquitin protein ligase 5	36.88	1.74E-09	2.05	0.005881914
MX2	MX dynamin like GTPase 2	31.78	1.72E-12	4.70	6.16E-10
EPSTI1	epithelial stromal interaction 1	30.52	9.02E-12	47.99	1.81E-12
IFI6	interferon alpha inducible protein 6	29.20	1.74E-11	4.18	4.74E-08
XAF1	XIAP associated factor 1	28.27	2.00E-10	38.85	5.22E-11
BST2	bone marrow stromal cell antigen 2	26.47	4.01E-11	19.49	5.40E-11
CMPK2	cytidine/uridine monophosphate kinase 2	25.81	5.92E-12	7.61	2.99E-10
XAF1	XIAP associated factor 1	25.27	5.28E-09	40.52	1.05E-09
IFI44	interferon induced protein 44	23.11	2.68E-12	17.21	2.49E-12
CXCL11	C-X-C motif chemokine ligand 11	22.68	3.83E-12	63.25	1.28E-13
IFIT2	interferon induced protein with tetratricopeptide repeats 2	20.65	4.62E-10	5.19	1.65E-07
OAS2	2'-5'-oligoadenylate synthetase 2	20.51	1.41E-09	6.34	1.63E-07
ISG15	ISG15 ubiquitin-like modifier	20.35	9.16E-10	3.73	3.26E-06
MX1	MX dynamin like GTPase 1	20.18	1.22E-10	6.40	1.05E-08
IFIT3	interferon induced protein with tetratricopeptide repeats 3	19.94	6.95E-12	11.86	2.29E-11
FRY	FRY microtubule binding protein	-7.72	3.84E-08	-1.90	0.002295319
G0S2	G0/G1 switch 2	-7.80	2.78E-10	-4.85	2.70E-09
LAMB2P1	laminin subunit beta 2 pseudogene 1	-7.94	8.11E-09	-1.70	0.003174398
WFDC2	WAP four-disulfide core domain 2	-7.94	1.12E-07	-6.79	2.51E-07
CREG2	cellular repressor of E1A stimulated genes 2	-8.05	1.91E-05	-10.75	6.88E-06
ELFN2	extracellular leucine rich repeat and fibronectin type III domain containing 2	-8.25	4.13E-07	-4.33	1.76E-05
CXCR4	C-X-C motif chemokine receptor 4	-9.36	7.61E-07	-5.10	2.04E-05
PPP1R3C	protein phosphatase 1 regulatory subunit 3C	-9.65	7.03E-08	-4.44	4.96E-06
KLK8	kallikrein related peptidase 8	-9.74	1.11E-09	-2.09	7.31E-05
S1PR4	sphingosine-1-phosphate receptor 4	-9.96	1.91E-09	-3.51	7.17E-07
PCP4L1	Purkinje cell protein 4 like 1	-10.12	4.29E-07	-2.73	0.001268127
HLA-DRB5	major histocompatibility complex, class II, DR beta 5	-10.24	6.12E-06	51.00	3.42E-08
SLC11A2	solute carrier family 11 member 2	-10.39	1.74E-09	-1.57	0.007465497
CAPN14	calpain 14	-10.40	1.16E-08	-1.98	0.001537628
LGALS14	galectin 14	-11.46	1.41E-08	3.47	1.19E-05
TNFSF4	TNF superfamily member 4	-12.12	9.70E-09	-3.28	1.65E-05
DIRAS2	DIRAS family GTPase 2	-12.39	1.85E-05	-13.08	1.93E-05
PRDM16	PR/SET domain 16	-14.33	4.22E-07	-2.25	0.015760879
LEMD1	LEM domain containing 1	-20.56	4.31E-09	2.33	0.000919216
KLK7	kallikrein related peptidase 7	-21.96	2.43E-11	-3.23	2.34E-07

Appendix Table S1: Twenty genes with the highest change of expression (increase and decrease) upon SARS-CoV-2 infection.

2) Appendix Table S2: Exact p-values

Fig 1E

Tukey's multiple comparisons test	Significant?	Summary	Adjusted P Value
FM vs. FM+IFN	No	ns	0,9863
FM vs. DIFF	No	ns	0,9723
FM vs. DIFF+IFN	Yes	***	0,0009
FM+IFN vs. DIFF	No	ns	0,9997
FM+IFN vs. DIFF+IFN	Yes	**	0,0013
DIFF vs. DIFF+IFN	Yes	**	0,0015
n=3			

Fig 1F

Tukey's multiple comparisons test	Significant?	Summary	Adjusted P Value
FM vs. FM+IFNg	No	ns	>0,9999
FM vs. DIFF	Yes	***	0,0003
FM vs. DIFF+IFNg	No	ns	0,0619
FM+IFNg vs. DIFF	Yes	***	0,0003
FM+IFNg vs. DIFF+IFNg	No	ns	0,063
DIFF vs. DIFF+IFNg	Yes	**	0,0094
n=3			

Fig 1G

Tukey's multiple comparisons test	Significant?	Summary	Adjusted P Value
FM vs. FM + IFN	No	ns	0,244
FM vs. DIff	Yes	****	<0,0001
FM vs. DIF + IFN	Yes	**	0,0032
FM + IFN vs. DIff	Yes	****	<0,0001
FM + IFN vs. DIF + IFN	Yes	***	0,0004
DIff vs. DIF + IFN	Yes	**	0,0047
n=3			

Fig 1H

Tukey's multiple comparisons test	Significant?	Summary	Adjusted P Value
FM vs. FM+IFN	Yes	****	<0,0001
FM vs. DIFF	Yes	****	<0,0001
FM vs. DIFF+IFN	Yes	****	<0,0001
FM+IFN vs. DIFF	Yes	*	0,0483
FM+IFN vs. DIFF+IFN	No	ns	0,0501
DIFF vs. DIFF+IFN	No	ns	>0,9999
n=3			

Fig 1I

Tukey's multiple comparisons test	Significant?	Summary	Adjusted P Value
FM vs. FM+IFN	No	ns	0,9998
FM vs. DIFF	No	ns	0,9288
FM vs. DIFF+IFN	Yes	*	0,0229
FM+IFN vs. DIFF	No	ns	0,9502
FM+IFN vs. DIFF+IFN	Yes	*	0,0254
DIFF vs. DIFF+IFN	No	ns	0,0529
n=3			

Fig 2B

Unpaired t test		
P value		0,0202
P value summary	*	
Significantly different (P < 0.05)?	Yes	
One- or two-tailed P value?	Two-tailed	
n=6		

Fgi 2C

Unpaired t test		
P value		0,0322
P value summary	*	
Significantly different (P < 0.05)?	Yes	
One- or two-tailed P value?	Two-tailed	
n=9		

Fig 2D

Unpaired t test		
P value		0,0133
P value summary	*	
Significantly different (P < 0.05)?	Yes	
One- or two-tailed P value?	Two-tailed	
n=4		

Fig 2E

Unpaired t test		
P value		0,0105
P value summary	*	
Significantly different (P < 0.05)?	Yes	
One- or two-tailed P value?	Two-tailed	
n=4		

Fig 2F right

Tukey's multiple comparisons test	Significant?	Summary	Adjusted P Value
DIFF vs. DIFF+IFN	Yes	*	0,0102
DIFF vs. DIFF 24h	No	ns	0,9629
DIFF vs. DIFF +IFN 24h	No	ns	0,9747
DIFF+IFN vs. DIFF 24h	Yes	*	0,0135
DIFF+IFN vs. DIFF +IFN 24h	Yes	*	0,0149
DIFF 24h vs. DIFF +IFN 24h	No	ns	>0,9999
n=4			

Fig 2F left

Tukey's multiple comparisons test	Significant?	Summary	Adjusted P Value
FM vs. FM +IFN	No	ns	0,0718
FM vs. FM 24h	No	ns	0,9526
FM vs. FM+IFN 24h	No	ns	0,9806
FM +IFN vs. FM 24h	No	ns	0,4904
FM +IFN vs. FM+IFN 24h	No	ns	0,5241
FM 24h vs. FM+IFN 24h	No	ns	0,9998
n=8			

Fig 2H

Tukey's multiple comparisons test	Significant?	Summary	Adjusted P Value
Diff vs. DIFF+IFN	Yes	*	0,0243
Diff vs. FM	No	ns	0,7419
Diff vs. FM+IFNg	No	ns	0,81
DIFF+IFN vs. FM	Yes	**	0,0042
DIFF+IFN vs. FM+IFNg	Yes	**	0,0028
FM vs. FM+IFNg	No	ns	0,9969

Fig 3E

Unpaired t test	
P value	<0,0001
P value summary	****
Significantly different (P < 0.05)?	Yes
One- or two-tailed P value?	Two-tailed
n=8	

Fig 3F

CXCL11		
Unpaired t test		
P value		0,0085
P value summary	**	
Significantly different (P < 0.05)?	Yes	
One- or two-tailed P value?	Two-tailed	
n=3		

IFIT3		
Unpaired t test		
P value		0,0232
P value summary	*	
Significantly different (P < 0.05)?	Yes	
One- or two-tailed P value?	Two-tailed	
n=3		

Krt20		
Unpaired t test		
P value		0,2386
P value summary	ns	
Significantly different (P < 0.05)?	No	
One- or two-tailed P value?	Two-tailed	
n=3		

ACE2		
Unpaired t test		
P value		0,0437
P value summary	*	
Significantly different (P < 0.05)?	Yes	
One- or two-tailed P value?	Two-tailed	
n=3		

EV1A left

Tukey's multiple comparisons test	Significant?	Summary	Adjusted P Value
FM vs. Diff	Yes	***	0,0001
FM vs. DIFF+IFN	Yes	****	<0,0001
FM vs. FM+IFN	No	ns	0,4063
Diff vs. DIFF+IFN	No	ns	0,9962
Diff vs. FM+IFN	Yes	**	0,0056
DIFF+IFN vs. FM+IFN	Yes	**	0,0027
DIFF n=7 and FM n=14			

EV1A right

Tukey's multiple comparisons test	Significant?	Summary	Adjusted P Value
FM vs. FM + IFN	Yes	**	0,0033
FM vs. DIff	No	ns	0,6959
FM vs. DIF + IFN	Yes	**	0,002
FM + IFN vs. DIff	Yes	*	0,0475
FM + IFN vs. DIF + IFN	No	ns	0,9969
DIff vs. DIF + IFN	Yes	*	0,03
n=8			

EV1B

ANOVA summary		
F		4,905
P value		0,0279
P value summary	*	
Significant diff. among means (P < 0.05)?	Yes	
R squared		0,8078
Brown-Forsythe test		
F (DFn, DFd)	7,966e+031 (6, 7)	
P value	<0,0001	
P value summary	****	
n=2		

EV1C

Tukey's multiple comparisons test	Significant?	Summary	Adjusted P Value
FM vs. FM+IFNg	Yes	****	<0,0001
FM vs. DIFF+IFN	Yes	****	<0,0001
FM vs. Diff	Yes	****	<0,0001
FM+IFNg vs. DIFF+IFN	Yes	****	<0,0001
FM+IFNg vs. Diff	No	ns	0,697
DIFF+IFN vs. Diff	Yes	****	<0,0001
n=124			

EV1E

left			
Tukey's multiple comparisons test	Significant?	Summary	Adjusted P Value
FM vs. FM + IFN	No	ns	>0,9999
FM vs. DIff	No	ns	0,0694
FM vs. DIF + IFN	Yes	**	0,0057
FM + IFN vs. DIff	Yes	*	0,0407
FM + IFN vs. DIF + IFN	Yes	**	0,0029
DIff vs. DIF + IFN	No	ns	0,1816
n=3			

EV1E

right			
Tukey's multiple comparisons test	Significant?	Summary	Adjusted P Value
FM vs. FM+IFNg	No	ns	0,6876
FM vs. DIFF	Yes	*	0,0158
FM vs. DIFF+IFNg	No	ns	0,499
FM+IFNg vs. DIFF	No	ns	0,0746
FM+IFNg vs. DIFF+IFNg	No	ns	0,9852
DIFF vs. DIFF+IFNg	No	ns	0,1208
n=3			

EV2F

left
Unpaired t test
P value
P value summary
Significantly different (P < 0.05)?
One- or two-tailed P value?
n=8

EV2F

right
Unpaired t test
P value
P value summary
Significantly different (P < 0.05)?
One- or two-tailed P value?
n=8

EV2E

Tukey's multiple comparisons test	Significant?	Summary	Adjusted P Value
control vs. -WC	Yes	****	<0,0001
control vs. Infg 3d	Yes	****	<0,0001
-WC vs. Infg 3d	No	ns	0,7724
n=9			

EV3A

Unpaired t test	
P value	<0,0001
P value summary	****
Significantly different (P < 0.05)?	Yes
One- or two-tailed P value?	Two-tailed
t, df	t=12,85, df=10
n=6	

EV4A

ACE2		
Unpaired t test		
P value		0,0027
P value summary	**	
Significantly different (P < 0.05)?	Yes	
One- or two-tailed P value?	Two-tailed	
n=7		

KRT20		
Unpaired t test		
P value		0,0028
P value summary	**	
Significantly different (P < 0.05)?	Yes	
One- or two-tailed P value?	Two-tailed	
n=7		

CXCL11		
Unpaired t test		
P value		0,0222
P value summary	*	
Significantly different (P < 0.05)?	Yes	
One- or two-tailed P value?	Two-tailed	
n=7		

IFIT3		
Unpaired t test		
P value		0,0025
P value summary	**	
Significantly different (P < 0.05)?	Yes	
One- or two-tailed P value?	Two-tailed	
n=7		
