# Supplementary material for: Epithelial response to IFN‐γ promotes SARS‐CoV‐2 infection
Source: EMBO Mol Med. 2021 Mar 3;13(4):e13191. doi: 10.15252/emmm.202013191 (PMC7995094; doi:10.15252/emmm.202013191)
Supplement: Supplementary file 2 — Expanded View Figures PDF [file EMMM-13-e13191-s003.pdf]

# Expanded View Figures

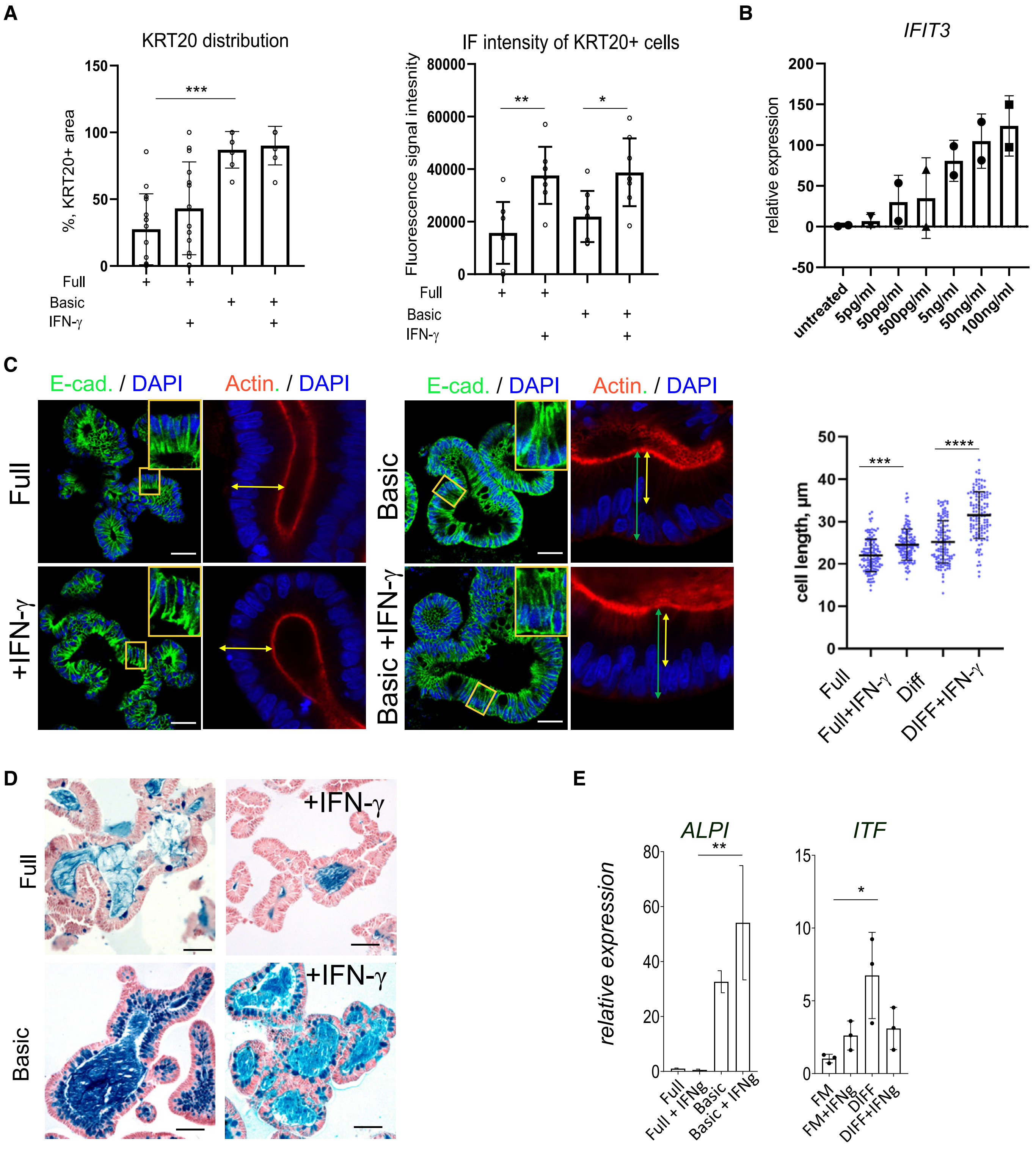


**Figure EV1.**

**Figure EV1. Differentiation of colon organoids after IFN-γ treatment.**

A Quantification of KRT20 immunofluorescence of human colon organoids cultured either in full or basic medium and treated with IFN-γ. Left: relative abundance of $KRT20^{+}$ cells per organoid; Right: Fluorescence intensity of $KRT20^{+}$ cells, indicating an increase in KRT20 expression in response to IFN-γ. Data are presented as mean ± SD, *: $P \leq 0.05$, **: $P \leq 0.001$, ***: $P \leq 0.0001$, as determined by one-way ANOVA, followed by Tukey's multiple comparisons test (more details see Appendix Table S2).

B Measurement of IFN-γ responsiveness by qRT–PCR for the IFN-γ target gene *IFIT3* at concentrations from 5 pg/ml to 100 ng/ml.

C Immunofluorescence staining for E-cadherin (green) and actin (red) of organoids grown in full medium (left) or basic medium (right) and additionally treated with IFN-γ (lower), indicating increased columnar cell height as a feature of differentiation upon IFN-γ treatment. Scale bar: 25 μm. Quantification of cell length on the right. Data are presented as mean ± SD, *: $P \leq 0.05$, **: $P \leq 0.001$, ***: $P \leq 0.0001$, as determined by one-way ANOVA, followed by Tukey's multiple comparison test (more details see Appendix Table S2).

D Alcian blue–nuclear fast red staining of human colon organoids cultured in full medium (upper panel) or differentiation medium (lower panel), with or without IFN-γ treatment to visualize goblet cells. Scale bar: 50 μm.

E Comparison of the differentiation markers ALPI (enterocytes, left) and ITF (goblet cells, right) in organoids grown in full medium or in basic medium and either untreated or treated with IFN-γ. Data are presented as mean ± SD, *: $P \leq 0.05$, **: $P \leq 0.001$, ***: $P \leq 0.0001$, as determined by one-way ANOVA followed by Tukey's multiple comparison test.

**Figure EV2. Interferon-γ (IFN-γ) induces differentiation into enterocytes and upregulates Ace2 expression in murine colon organoids.**

A Immunofluorescence for Ace2 (red) and Krt20 (green) in murine colon: pronounced expression of *Ace2* in terminally differentiated surface enterocytes at the top of the colon crypt. Magnifications of single fluorescence channels below. Scale bar: 50 μm.

B t-sne plot for selected genes from single-cell RNAseq of mouse colon revealing expression of *Krt20* in enterocytes ($Car4^{+}$ population) and partially in goblet cells ($Tff3^{+}$ population). Interferon receptors (Ifngr1/2) show rather broad expression in the mouse colon epithelium.

C 3-dimensional organoid culture from mouse colon epithelium using different culture conditions; upper: full medium (Full), middle: upon Wnt withdrawal (-Wnt, -CHIR (-WC)), lower: FM supplemented with IFN -γ (Ifn-γ). Immunofluorescence for Krt20 (green) middle panel, and immunofluorescence for E-cadherin (green), third from right, and phalloidin staining for actin (right) Scale bar: 50 μm. Magnification of IFN-γ-treated organoids stained for Krt20 and E-cadherin below.

D Western blot of protein lysates from mouse colon organoids cultured in full medium or treated with IFN-γ, showing increase of Krt20 protein upon IFN-γ treatment. Tubulin served as loading control.

E Optical section of whole mount immunofluorescence of 2 h EdU incorporation (red) in organoids cultured in full medium, -WC medium or treated with IFN-γ to label proliferating cells in s-phase. Right: quantification of EdU-positive nuclei as percentage of all nuclei per organoid. Data are presented as mean ± SD, *: $P \leq 0.05$, **: $P \leq 0.001$, ***: $P \leq 0.0001$, as determined by one-way ANOVA, followed by Tukey's multiple comparison test (more details see Appendix Table S2).

F Comparison of *Krt20* mRNA expression of control (full medium) and IFN-γ-treated organoids, as determined by qPCR. Data are presented as mean ± SD *: $P \leq 0.05$, **: $P \leq 0.001$, as determined by Student's *t*-test (more details see Appendix Table S2).

 © 2021 The Authors

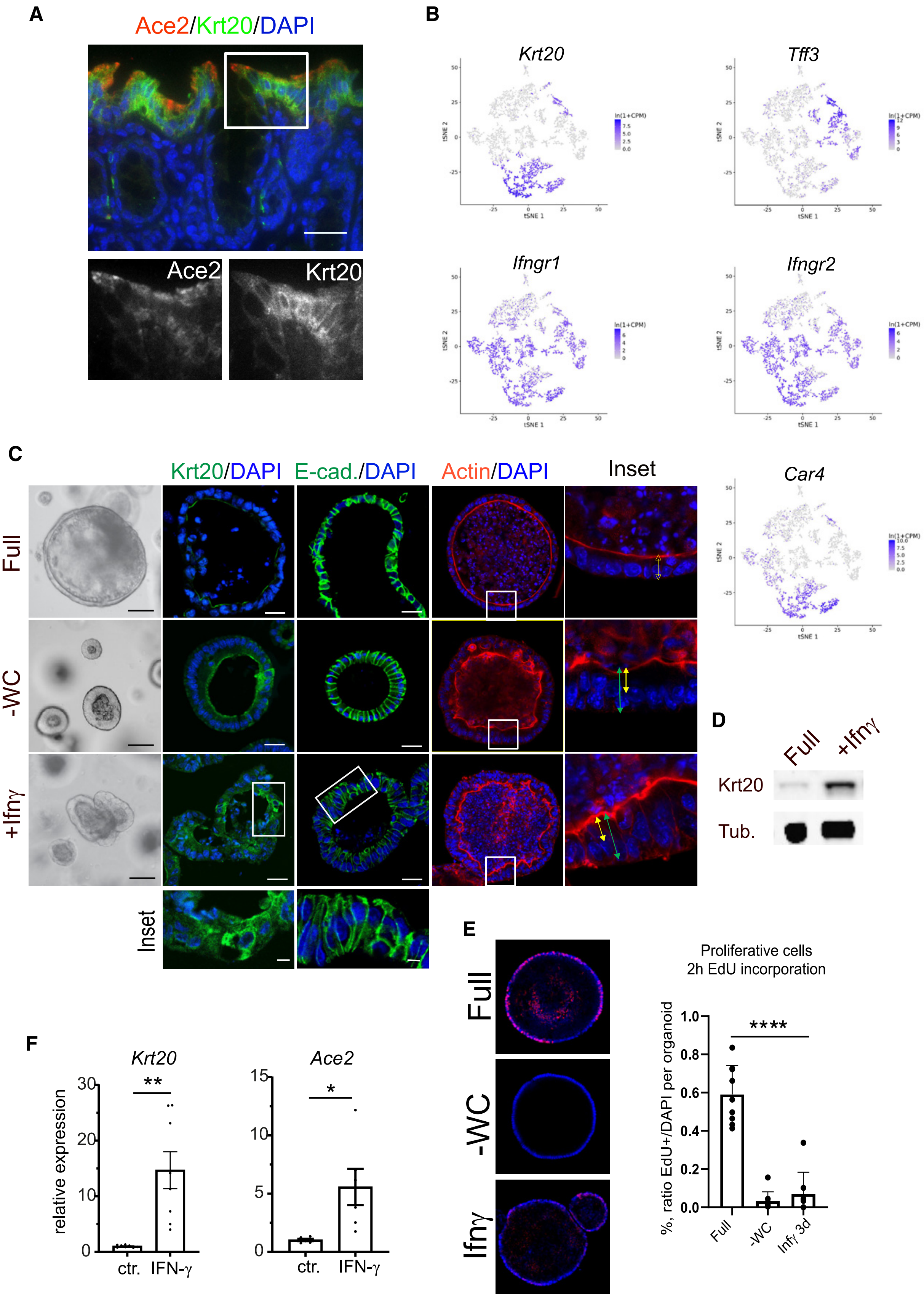
A
Ace2/Krt20/DAPI
Ace2
Krt20
B
Krt20
Tff3
Ifngr1
Ifngr2
Car4
C
Krt20/DAPI
E-cad./DAPI
Actin/DAPI
Inset
Full
-WC
+Ifnγ
Inset
D
Full
+Ifnγ
Krt20
Tub.
E
Full
-WC
Ifnγ
Proliferative cells
2h EdU incorporation
%, ratio EdU+/DAPI per organoid
****
Full
-WC
Infγ 3d
F
Krt20
Ace2
relative expression
**
*
ctr.
IFN-γ

**Figure EV2.**

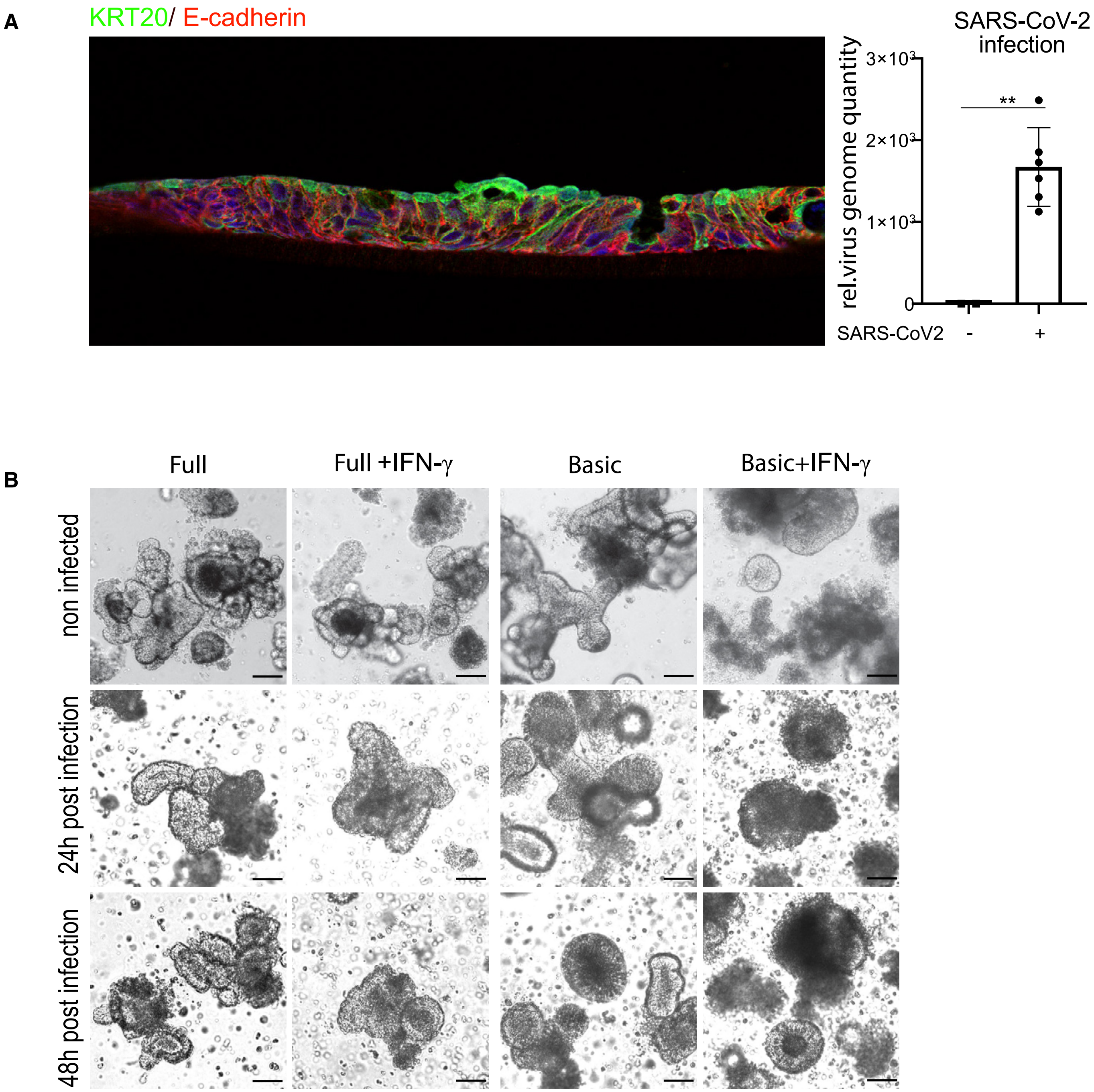


**Figure EV3. Infection of air–liquid interface culture and tracking of SARS-CoV-2-infected organoids.**

A Air–liquid interface culture of human colonocytes. Immunofluorescence for KRT20 (green) and E-cadherin (red), indicating differentiated epithelial cells. Right: qPCR data displaying the relative virus load of SARS-CoV-2 measured by viral genome quantity in ALI cells, normalized to GAPDH. Data are presented as mean ± SD, **: $P \leq 0.01$, as determined by Student's *t*-test (more details see Appendix Table S2).

B Bright field images of human colon organoids cultured in full medium (left), full medium + IFN-γ (second right), differentiated (third right) and differentiated + IFN-γ (far right), either uninfected (upper panel), or after 24 h (middle panel) or 48 h (lower panel) post-infection with SARS-CoV-2. Scale bar: 50 μm.

 © 2021 The Authors

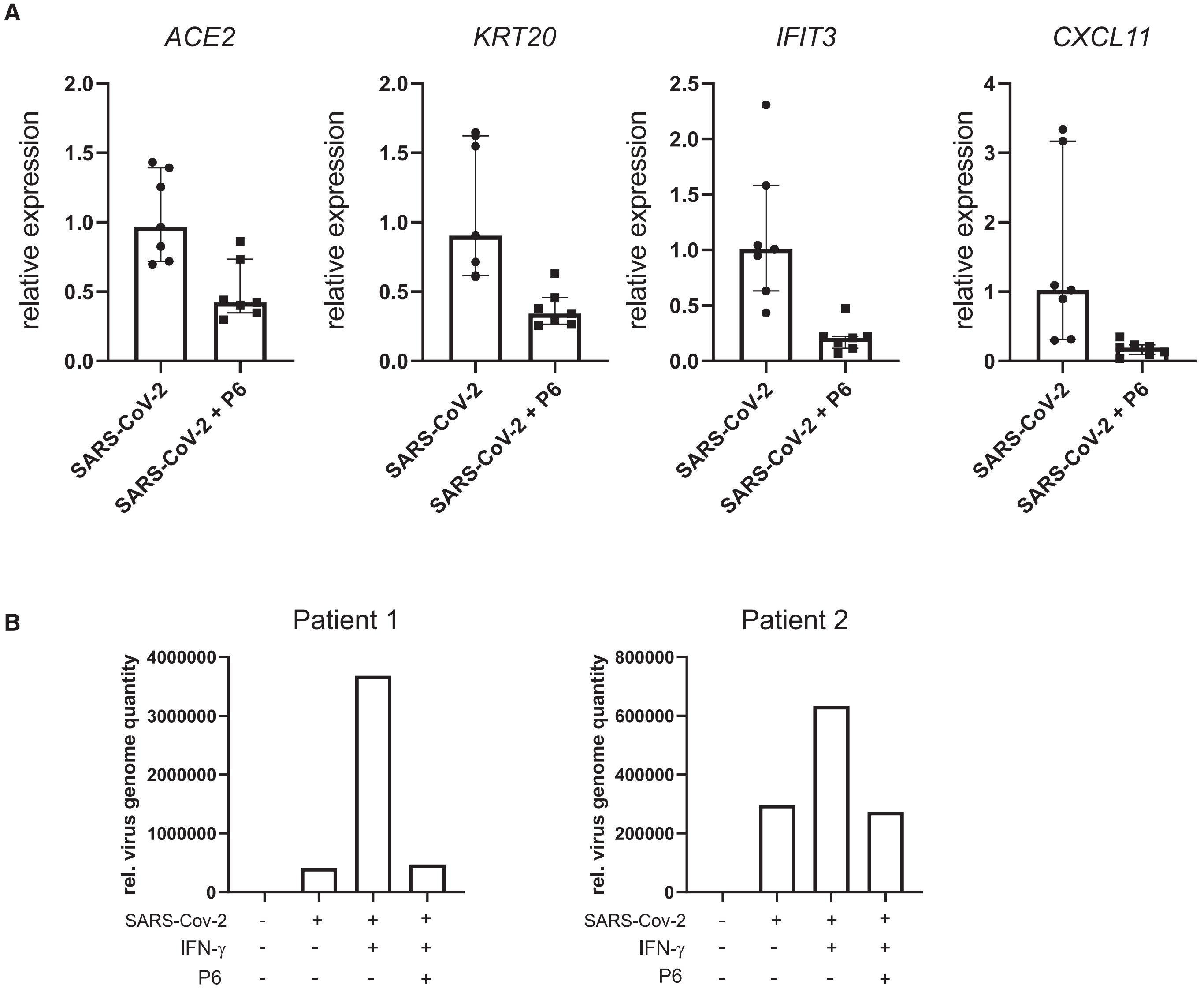


**Figure EV4. Analysis of pan-JAK inhibition upon IFN-γ treatment and SARS-CoV-2 infection.**

A qPCR data of differentiated and SARS-CoV-2-infected human colon organoids treated with the pan-JAK inhibitor pyridone-6 (P6). Data generated from four biological replicates from two patients. Median with interquartile range. Data indicating decreased expression of *ACE2*, *KRT20*, *CXCL11*, and *IFIT3 upon* P6 treatement. Data are presented as mean ± SD, *: $P \leq 0.05$, **: $P \leq 0.001$, ***: $P \leq 0.0001$, as determined by Student's *t*-test (more details see Appendix Table S2).

B qPCR data comparing the relative virus load of SARS-CoV-2 measured by viral genome quantity normalized to *GAPDH* at 48 h after infection in basic medium untreated and treated with IFN-γ and pyridone-6, indicating that IFN-γ increases virus load, which is reduced upon inhibition of JAK kinases. Data assessed from two different patient samples are shown.
